# Genomic and ecological systems-thinking framework for pathogenic *Leptospira* in Puerto Rico

**DOI:** 10.3389/fpubh.2026.1838089

**Published:** 2026-08-31

**Authors:** Ammar Yasir, Gillian A. M. Tarr, Claudia Munoz-Zanzi

**Affiliations:** Division of Environmental Health Sciences, School of Public Health, University of Minnesota, Minneapolis, MN, United States

**Keywords:** ecology, genomic epidemiology, *Leptospira borgpetersenii*, *Leptospira interrogans*, *Leptospira kirschneri*, SNP threshold calibration, spatiotemporal analysis, systems thinking

## Abstract

**Introduction:**

Leptospirosis is a complex zoonotic disease requiring high-resolution surveillance. A systems-thinking framework was used to connect genomic and ecological data and map the geographic and host-based structuring of co-circulating pathogenic *Leptospira* lineages in Puerto Rico.

**Methods:**

Forty-four core genomes of *L. interrogans*, *L. borgpetersenii*, and *L. kirschneri* from human, domestic, and wildlife hosts were analyzed. Spatiotemporal and landscape metadata were integrated using root-to-tip regression, isolation-by-distance profiling and calibrated single-nucleotide polymorphism (SNP) thresholds (≤1, ≤5, and ≤10 SNPs) to define transmission clusters.

**Results:**

*Leptospira* species exhibited distinct ecological pathways partitioned by geography, explaining 56% of genomic variance for *L. interrogans* and 91% for *L. borgpetersenii* (PERMANOVA). *L. interrogans* displayed high landscape connectivity across multiple hosts, forming localized networks (≤1 to ≤10 SNPs) that capture active spillovers (human-to-rat linkages at ≤1 SNP) and resolved into rodent host-specific lineages (*R^2^* = 0.34). Conversely, *L. borgpetersenii* showed spatial and temporal genomic homogeneity and a lack of host-associated structure within an unpartitioned transmission pool dominated by *Mus musculus*. As a result, fixed genomic thresholds yielded disparate outcomes: *L. interrogans* resolved into 4 to 5 discrete, expanding clusters, whereas *L. borgpetersenii* grouped into a single uniform population at the ≤10-SNP threshold.

**Conclusion:**

Co-circulating pathogenic leptospires occupy distinct ecological niches shaped by varying host restriction and environmental persistence. Fixed genomic thresholds lack universal applicability; effective genomic epidemiological surveillance must employ species-specific threshold calibration to accurately map transmission pathways.

## 1 Introduction

Leptospirosis, a globally significant zoonotic disease caused by spirochetes of the genus *Leptospira*, accounts for over one million clinical cases and 58,900 deaths annually (1). Its transmission ecology is driven by a complex interplay of environmental factors, diverse animal hosts, and the genomic diversity of *Leptospira* species (2). *Leptospira* species are classified into two major clades: saprophytic (S) and pathogenic (P) (3). These clades are further sub-classified into subclades S1 and S2 (26 and 5 species, respectively) and P1 and P2 (20 and 21 species, respectively). Subclade P1 includes high- (P1^+^) and low-virulence (P1^−^) pathogens, while P2 consists of species with intermediate virulence (3).

The ecology of *Leptospira* involves a spectrum of maintenance hosts (predominantly rodents sustaining chronic, asymptomatic infections) and incidental hosts, including humans, who typically develop acute infections without long-term pathogen shedding (4). Historically, attempts to identify infection sources relied on assumptions of serovar host adaptation (5). However, cross-species serovar sharing and labor-intensive isolate serotyping complicate efforts to infer transmission pathways (6, 7). Analysis of Whole Genome Sequences (WGS) helps to overcome these barriers by utilizing core-genome Single Nucleotide Polymorphism (SNP) alignments to delineate high-resolution transmission clusters and cross-species spillover events (8, 9).

Despite these advances, genomic epidemiological studies of *Leptospira* remain limited due to unavailable or incomplete metadata, low numbers of sequences from a given location and time, and the inherent diagnostic challenges. Prior *Leptospira* genomic studies investigated single host species in an urban temperate climate (10) and a specific outbreak (11). The genomic diversity and spatiotemporal clustering of *Leptospira* species circulating sympatrically in endemic tropical regions remain underexplored.

This study adopts a systems-thinking framework (12) to analyze pathogenic *Leptospira* genomes from Puerto Rico. Recognizing that surveillance gaps often constrain the traditional reconstruction of linear transmission chains, we instead interpret core-genome structure as a direct realization of ecological connectivity shaped by host distributions and landscape organization. To achieve this, we used cross-cohort sequences across varying SNP thresholds. By analyzing micro-evolutionary distances alongside discrete spatial coordinates and host metadata, this study aims to: (1) map the multi-species landscape partitioning and host-based structuring of *Leptospira* diversity; (2) identify spatiotemporally linked transmission clusters using calibrated genomic thresholds; and (3) bridge the gap between genomic connectivity and landscape-scale ecological spread. Ultimately, this work contributes new insight into how pathogenic lineages persist and cross species boundaries in a highly connected tropical environment.

## 2 Materials and methods

### 2.1 Study area, data retrieval, and quality control

**Figure 1:**
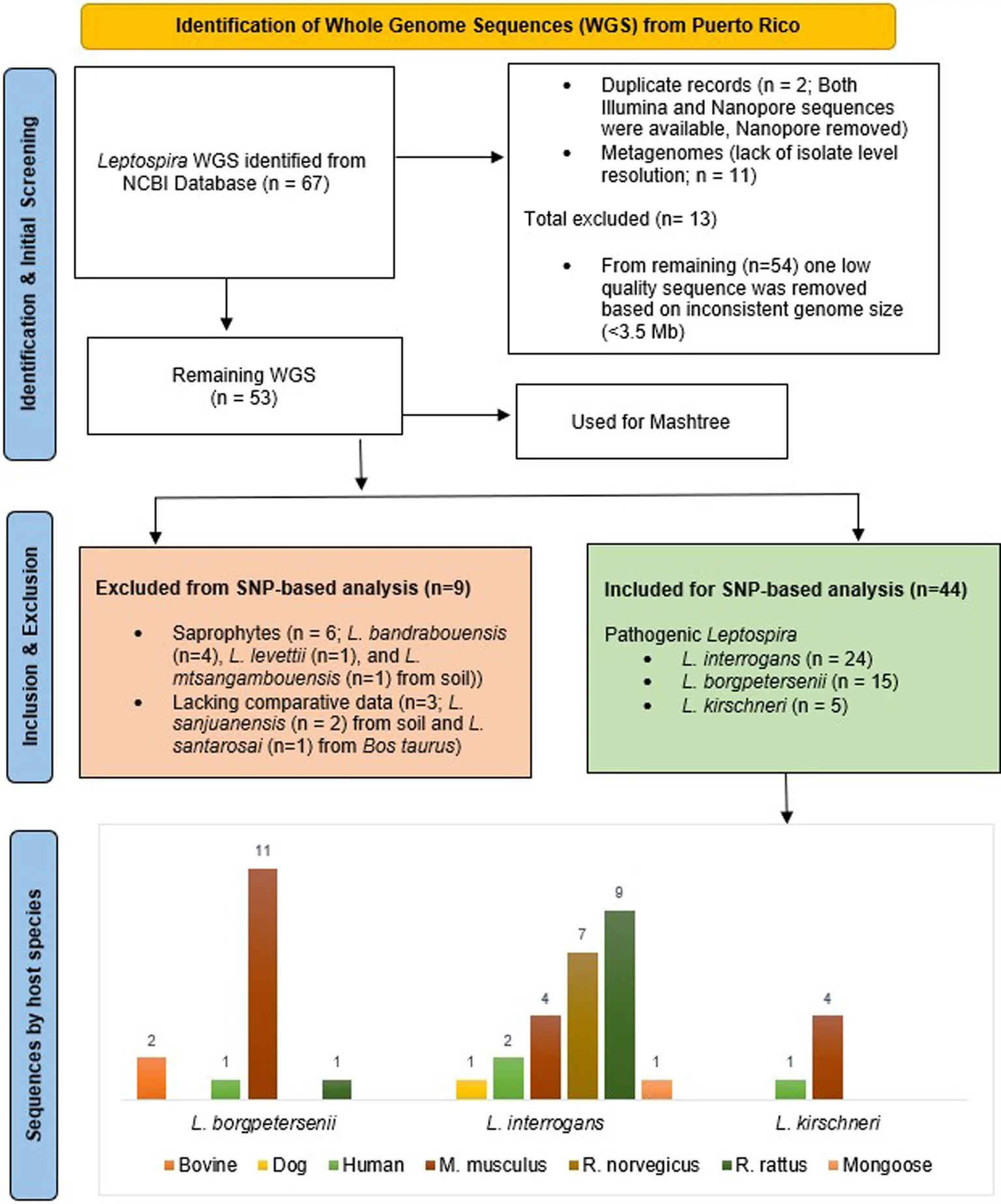
Flowchart showing the identification, quality control, and selection of *Leptospira* whole-genome sequences from Puerto Rico obtained from NCBI (2015–2021). Inclusion and exclusion criteria yielded a final dataset of 44 genomes for core-genome SNP-based analysis. Distribution of the final 44 genomes analyzed by *Leptospira* species and host species is presented by a bar graph.

Puerto Rico’s tropical, flood-prone climate facilitates endemic *Leptospira* transmission across human and animal populations (13–15). WGS metadata for 67 local isolates from previous studies (13–16) were screened from the National Center for Biotechnology Information (NCBI) database (Figure 1). The projects that originated the animal isolates were approved by the Institutional Care and Use Committees as described in the respective publications (14, 16). The human isolates were obtained as part of a public health surveillance project determined to be non-human subjects research by CDC’s National Center for Emerging and Zoonotic Infectious Diseases’ Human Subjects Advisor (Determination #031521IS) (13). Metagenomes (*n* = 11) and duplicate records (*n* = 2) were excluded. Raw reads for the remaining 54 isolates were retrieved via the SRA Toolkit v3.1.1 (Supplementary Table S1) and processed through Bactopia v3.1.0 (17). Quality control and adapter trimming were completed using FastQC v0.12.1 and Trimmomatic.

Genomes were assembled using Shovill v1.1.0 and evaluated against five quality metrics: contig count ≤400, N_50_ > 30 kb, average coverage > 30x, reference mapping rate > 90%, and total genome size (3.5–6.0 Mb). One assembly was excluded due to an inconsistent genome size. A distance dendrogram for the remaining 53 isolates was generated via the Bactopia Mashtree workflow (18) (Supplementary Figure S1). Six saprophytic genomes and three genomes lacking multi-host metadata were removed (Figure 1). Retained assemblies averaged an N_50_ of 77.4 kb with a median of 112.5 contigs.

The final dataset for SNP analyses included *L. interrogans* (*n* = 24), *L. borgpetersenii* (*n* = 15), and *L. kirschneri* (*n* = 5). Sequences were annotated with a unique identifier combining sampling site (range: 1–13), host species (B: bovine, D: dog, H: human, M: mongoose, MM: *Mus musculus*, RR: *Rattus rattus*, RN: *Rattus norvegicus*), and a numeric suffix indicating the isolate number at that site (range: 1–12). Sampling ZIP codes were mapped to 2024 US Census Bureau ZIP Code Tabulation Area (ZCTA) internal centroids (19).

### 2.2 Core genome phylogenomics and temporal signal audits

Core genome SNP alignments were generated for each *Leptospira* species using the Bactopia Snippy v4.6.0 workflow (Supplementary Table S2) against NCBI reference strains Fiocruz L1-130 (*L. interrogans*), Piyasena (*L. borgpetersenii*), and 3,522\u00B0C-T (*L. kirschneri*). Recombinant regions were removed using Gubbins v3.3.0, and maximum likelihood phylogenies were inferred using IQ-Tree v2.2.2.7 (Supplementary Figure S2). Core SNPs were extracted using snp-sites v2.5.1 (20), and pairwise distance matrices were calculated using PairSNP v0.3.1.

To evaluate whether phylogenetic trees were suitable for time-calibrated phylogenomic inference, root-to-tip regressions were performed using Clockor2 v1.10.2 (21). To identify potential leverage artifacts caused by single historical timepoints, models were cross-validated across two distinct temporal horizons using diagnostic sensitivity testing (22): a macro-timeline (all isolates) and a high-resolution micro-timeline restricted to active 2020–2021 isolates (*n* = 42).

### 2.3 Spatial structuring and geographic distance decay

Pairwise geographic distances between ZCTA internal centroids were calculated using the Haversine formula, which measures the direct distance between two points on a sphere using their coordinates (23). For *L. interrogans*, metrics were resolved into three spatial tiers based on geographic thresholds: local (<5 km), regional (5–20 km), and island-wide (>20 km). These distance thresholds broadly correspond to pairs located within the same ZCTA, within contiguous ZCTAs, and across non-contiguous ZCTAs, respectively. Conversely, the geographic distribution of sampled *L. borgpetersenii* isolates dictated a binary framework: local (<5 km) versus island-wide (>20 km) due to a spatial sampling gap between collection sites.

A Mantel Test (24) evaluated isolation-by-distance patterns by correlating genomic distances against geographic distance matrices. Spatial trends were visualized using a linear regression trend line for *L. interrogans* to capture its continuous spatial decay profile, and a local polynomial regression fitting (LOESS) (25) for *L. borgpetersenii* to accommodate a non-linear trajectory. To validate the robustness of these metrics, a post-hoc leave-one-out jackknife sensitivity analysis was executed across both datasets by iteratively omitting single isolates and recalculating Mantel statistics (26). Spatial variation across landscape boundaries was evaluated using distance-based permutational multivariate analysis of variance (PERMANOVA) calculated from a Euclidean distance matrix of pairwise core-genome SNP distances with 9,999 permutations, blocked by collection year, alongside multivariate homogeneity of groups dispersions analysis via PERMDISP (27). To quantify genomic effect sizes across spatial tiers, Hodges-Lehmann (HL) median difference contrasts with 95% confidence intervals (CIs) were computed (28). Finally, the proportion of epidemiologically linked isolate pairs matching the same ZCTA was tracked across three genomic thresholds (≤1, ≤5, and ≤10 SNPs) utilizing 95% Wilson CIs. These analyses focused on *L. interrogans* and *L. borgpetersenii* due to the limited sample size of *L. kirschneri*.

### 2.4 Genomic clustering and recent transmission linkage

Predefined genomic thresholds (≤1, ≤5, and ≤10 SNPs) were applied to the pairwise core genome SNP distance matrices using GraphSNP (29) to identify clustering patterns and integrate sampling site and host metadata. If no clusters were detected at these specific thresholds, values were increased incrementally by single SNPs in exploratory runs until networks were resolved. Transmission network configurations and minimum-distance links among expanding clusters and singletons were mapped via minimum spanning trees (MSTs) constructed using Kruskal’s algorithm (29).

Temporal, spatial, and host demographic characteristics were examined within each cluster. Epidemiological clustering analysis focused primarily on the contemporary 2020–2021 window (*n* = 253 pairs), utilizing historical isolates from 2015 and 2018 (*n* = 23 baseline pairs) to anchor genomic context and track baseline stability over time. Cluster-level spatial footprints were classified as “Same” if all pairs fell within a single ZCTA; “Contiguous” if all pairs occurred within adjacent, shared-boundary or vertex ZCTAs; and “Distant” if at least one pair mapped to non-contiguous ZCTAs. Clusters holding a “Same” or “Contiguous” profile during 2020–2021 were defined as spatiotemporal transmission chains.

### 2.5 Host associated and rodent specific genomic structuring

Host-driven genomic signatures for *L. interrogans* were visualized via principal coordinate analysis (PCoA) and evaluated using a distance-based PERMANOVA and PERMDISP framework (9,999 permutations, blocked by collection year) to partition Euclidean core-genome SNP distance matrices across all host species. Rodent-driven genomic structuring was analyzed using a subset matrix restricted to *L. interrogans* isolates from the three rodent species (*M. musculus*, *R. norvegicus*, and *R. rattus*; *n* = 20). Within this rodent network, HL median contrasts were calculated to quantify pairwise genomic distance shifts between distinct rodent species and compare intra-species versus inter-species genomic distances. Additionally, rodent demographic characteristics (age class) and sex were overlaid to explore host-related patterns within specific transmission chains. An identical workflow was deployed for *L. borgpetersenii* to contrast host-dependent structuring against a baseline of host-independent spatial and temporal genomic homogeneity. All statistical tests were executed in R version 4.6.0 using the vegan, geosphere, and tidyverse packages.

## 3 Results

### 3.1 Dataset baseline and sympatric population structure

**Figure 2:**
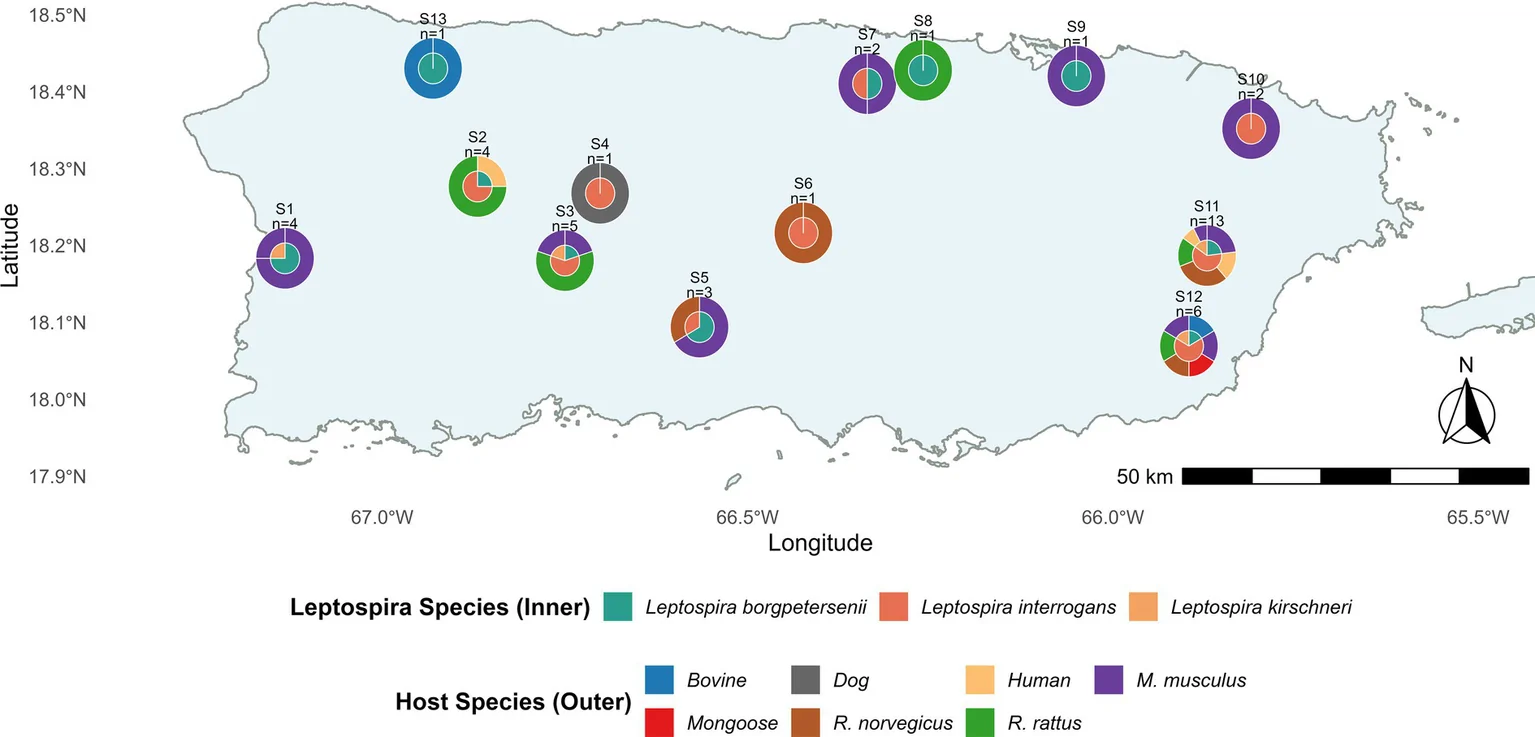
Geographic distribution of pathogenic *Leptospira* species and their corresponding hosts. The map of Puerto Rico shows sampling sites (S1-S13) and the total number of whole-genome sequences (*n*) obtained from isolates collected between 2015 and 2021 at each site. Nested donut charts at each site display the proportion of *Leptospira* species (inner circle) aligned with their matched host species (outer circle) to visualize sympatric host-pathogen correspondence.

High-resolution core-genome SNP analysis was performed on 44 pathogenic *Leptospira* sequences spanning 13 geographic sampling sites (1–13 sequences per site; Figure 2). Spatial mapping revealed sympatric host overlaps, multi-species co-circulation within individual ZCTA boundaries, and co-occurrence between human spillovers and local rodent reservoirs.

The sample set comprised *M. musculus* (*n* = 19), *R. rattus* (*n* = 10), *R. norvegicus* (*n* = 7), clinical humans (*n* = 4), mongoose (*n* = 1), and a dog (*n* = 1). Lineage specification grouped these isolates into *L. interrogans* (*n* = 24), *L. borgpetersenii* (*n* = 15, including two divergent bovine lineages), and *L. kirschneri* (*n* = 5). Detailed host demographic baselines are compiled in Supplementary Table S3.

### 3.2 Root-to-tip regression analysis and evolutionary dynamics

Root-to-tip regression optimization revealed distinct temporal signals and contrasting micro-evolutionary pathways among the three co-circulating *Leptospira* species (Supplementary Figure S3). *L. interrogans* showed a linear temporal signal across the macro-timeline (*R*^2^ = 0.23, substitution rate = 3.466 × 10^−2^ substitutions/site/year). This association strengthened when restricted to the contemporary micro-timeline (*R*^2^ = 0.30), with an accelerated molecular clock rate (5.55 × 10^−1^ substitutions/site/year). Conversely, *L. borgpetersenii* showed a linear progression across the multi-year macro-timeline (*R*^2^ = 0.24, rate = 1.22 × 10^−4^ substitutions/site/year), supported by its historical anchor isolate. Under micro-timeline sensitivity analysis, this contemporary temporal signal collapsed (*R*^2^ = 0.00, rate = −5.60 × 10^−5^ substitutions/site/year), confirming short-term temporal genomic homogeneity within its local host niche. *L. kirschneri* exhibited a synchronized evolutionary clock within the contemporary window (*R*^2^ = 0.62, rat = 4.50 × 10^−3^ substitutions/site/year).

### 3.3 *Leptospira interrogans* genomic results

#### 3.3.1 Core genomic profile and geographic structuring

**Figure 3:**
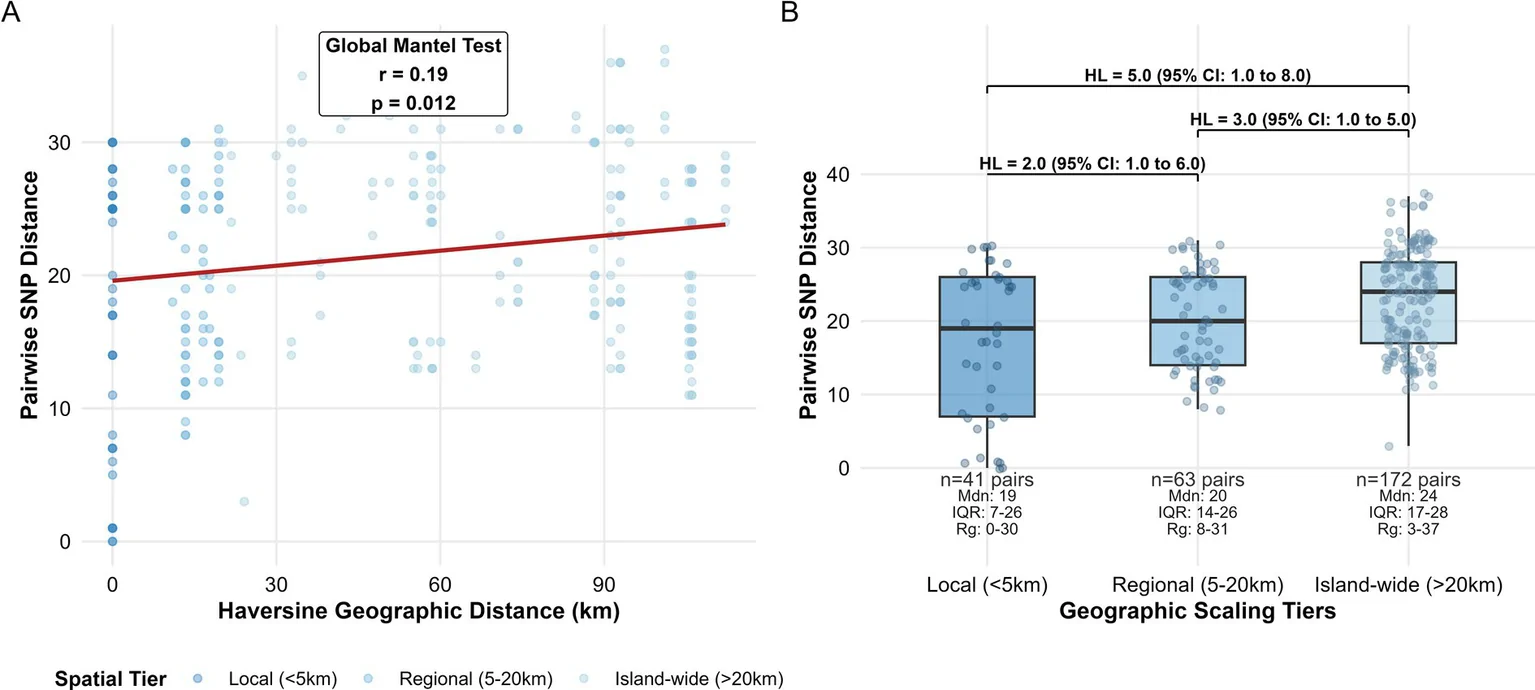
Spatial decay and micro-geographic structuring of *Leptospira interrogans* core genomes. **(A)** Absolute isolation-by-distance decay plot mapping pairwise core SNP distances against continuous Haversine geographic distances (km), overlaid with a linear regression trend line and global Mantel test statistics (*r* = 0.19, *p* = 0.01). Individual data points are color-coded by their corresponding spatial tier. **(B)** Pairwise core-genome SNP distances stratified across three geographic distance tiers: Local (<5 km; *n* = 41 pairs), regional (5–20 km; *n* = 63 pairs), and island-wide (>20 km; *n* = 172 pairs). Text overlays along the x-axis display group-specific descriptive statistics (median [mdn], IQR, and range [rg]). Horizontal brackets indicate calculated Hodges-Lehmann (HL) median difference effect sizes with their respective 95% confidence intervals.

The 24 *L. interrogans* genomes exhibited a median pairwise distance of 24 SNPs (IQR: 15–28, range: 0–37; Supplementary Figure S4), partitioned by geography. Distance-based PERMANOVA confirmed that sampling ZCTAs explained 56% of total genomic variance (*F* = 2.39; *p* = 0.002), with homogeneous group dispersions (PERMDISP *p* = 0.45). A Mantel test confirmed an isolation-by-distance pattern (r = 0.19, *p* = 0.01; Figure 3A), which remained robust under post-hoc jackknife sensitivity analysis (maximum *p* ≤ 0.04).

Evaluation across geographic tiers indicated a spatial decay gradient (Figure 3B). Local pairs (<5 km) had a median of 19 SNPs (IQR: 7–26), regional pairs (5–20 km) had a median of 20 SNPs (IQR: 14–26), and island-wide pairs (>20 km) reached a median of 24 SNPs (IQR: 17–28). HL contrasts confirmed that local isolates were shifted closer by a median of 5 SNPs (95% CI: 1–8) relative to island-wide pairs, while regional isolates were shifted closer by a median of 3 SNPs (95% CI: 1–5). Geographic tracking fidelity was high: at the stringent ≤1-SNP threshold, 6/6 (100%; 95% CI: 61–100) of pairs were co-located within the same ZCTA, decreasing to 14/16 (88%; 95% CI: 64–97) at ≤5 SNPs, and 18/24 (75%; 95% CI: 55–88) at ≤10 SNPs.

#### 3.3.2 SNP-based genomic clusters and spatiotemporal linkages

**Figure 4:**
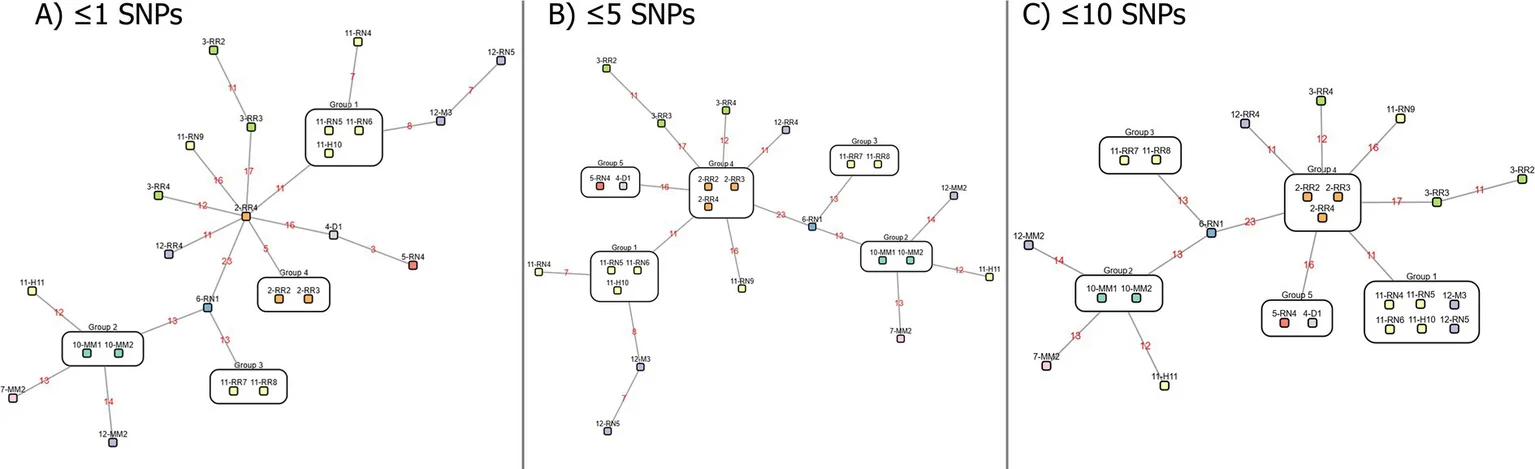
Minimum spanning trees (MSTs) of *Leptospira interrogans* isolates demonstrating genomic clustering behavior across fixed thresholds of **(A)** ≤ 1, **(B)** ≤ 5, and **(C)** ≤ 10 SNPs. Nodes are color-coded by geographic sampling site and labeled with their unique isolate identifier (denoting sampling site, host species [RN (*Rattus norvegicus*), MM (*Mus musculus*), RR (*Rattus rattus*), M (mongoose), H (human), and D (dog)], and isolate number). Edge labels and red numbers indicate absolute pairwise SNP distances between connected nodes.

Fixed genomic thresholds of ≤1, ≤5, and ≤10 SNPs resolved 4, 5, and 5 distinct clusters, respectively (Figure 4; Table 1). At the ≤1-SNP and ≤5-SNP thresholds, clusters were localized, containing 2 to 3 isolates from identical collection sites during 2021, and host species were homogeneous except for Group 1. Within Group 1, a human clinical case (11-H10, March 2021) clustered in near-identity (0–1 SNP divergence) with two local *R. norvegicus* isolates (11-RN5 and 11-RN6, May 2021) at the same site. At the ≤5-SNP threshold, Group 4 expanded to include an additional *R. rattus* isolate, while a historical 2018 anchor isolate from a domestic dog formed a cross-year, trans-species link with a 2021 *R. norvegicus* isolate in Group 5, bridging distinct hosts collected from physically distant collection sites (Table 1; Supplementary Figure S5). At ≤10 SNPs, clusters ranged from 2 to 6 isolates; Group 1 expanded to include *R. norvegicus* and mongoose isolates, shifting its classification to a Contiguous footprint (Table 1; Figure 4). All clusters showed spatiotemporal linkages except for Group 5. Notably, isolates collected from the same collection site did not always group into a single genomic cluster. Among the five sites where multiple isolates were collected, only site S10 (all thresholds) and site S2 (higher thresholds) were restricted to a single genomic cluster.

**Table 1:** Cluster groupings and spatiotemporal and host demographics characteristics of *L. interrogans* isolates at SNP thresholds of ≤1, ≤5, and ≤10.

Cluster ID	Isolate ID	Collection date	Host species	Sex/Age	Spatial classification
≤1 SNP Threshold					
Group 1	11-RN5	May 2021	*R. norvegicus*	Male/Adult	Same
	11-RN6	May 2021	*R. norvegicus*	Female/Adult	
	11-H10	March 2021	Human	-	
Group 2	10-MM1	May 2021	*M. musculus*	Male/Adult	Same
	10-MM2	May 2021	*M. musculus*	Female/Adult	
Group 3	11-RR7	May 2021	*R. rattus*	Male/Adult	Same
	11-RR8	May 2021	*R. rattus*	Female/Juvenile	
Group 4	2-RR2	April 2021	*R. rattus*	Male/Adult	Same
	2-RR3	April 2021	*R. rattus*	Female/Adult	
≤5 SNP Threshold					
(Groups 1–3)	Same as ≤1 SNP				
Group 4	2-RR2	April 2021	*R. rattus*	Male/Adult	Same
	2-RR3	April 2021	*R. rattus*	Female/Adult	
	2-RR4	April 2021	*R. rattus*	Female/Adult	
Group 5	5-RN4	April 2021	*R. norvegicus*	Male/Adult	Distant
	4-D1	2018	Dog	-	
≤10 SNP Threshold					
Group 1	11-RN5	May 2021	*R. norvegicus*	Male/Adult	Contiguous
	11-RN6	May 2021	*R. norvegicus*	Female/Adult	
	11-H10	March 2021	Human	-	
	11-RN4	May 2021	*R. norvegicus*	Male/Adult	
	12-M3	May 2021	Mongoose	Male/Adult	
	12-RN5	May 2021	*R. norvegicus*	Male/Adult	
(Groups 2–5)	Same as ≤5 SNP				

#### 3.3.3 Host-associated genomic structuring

**Figure 5:**
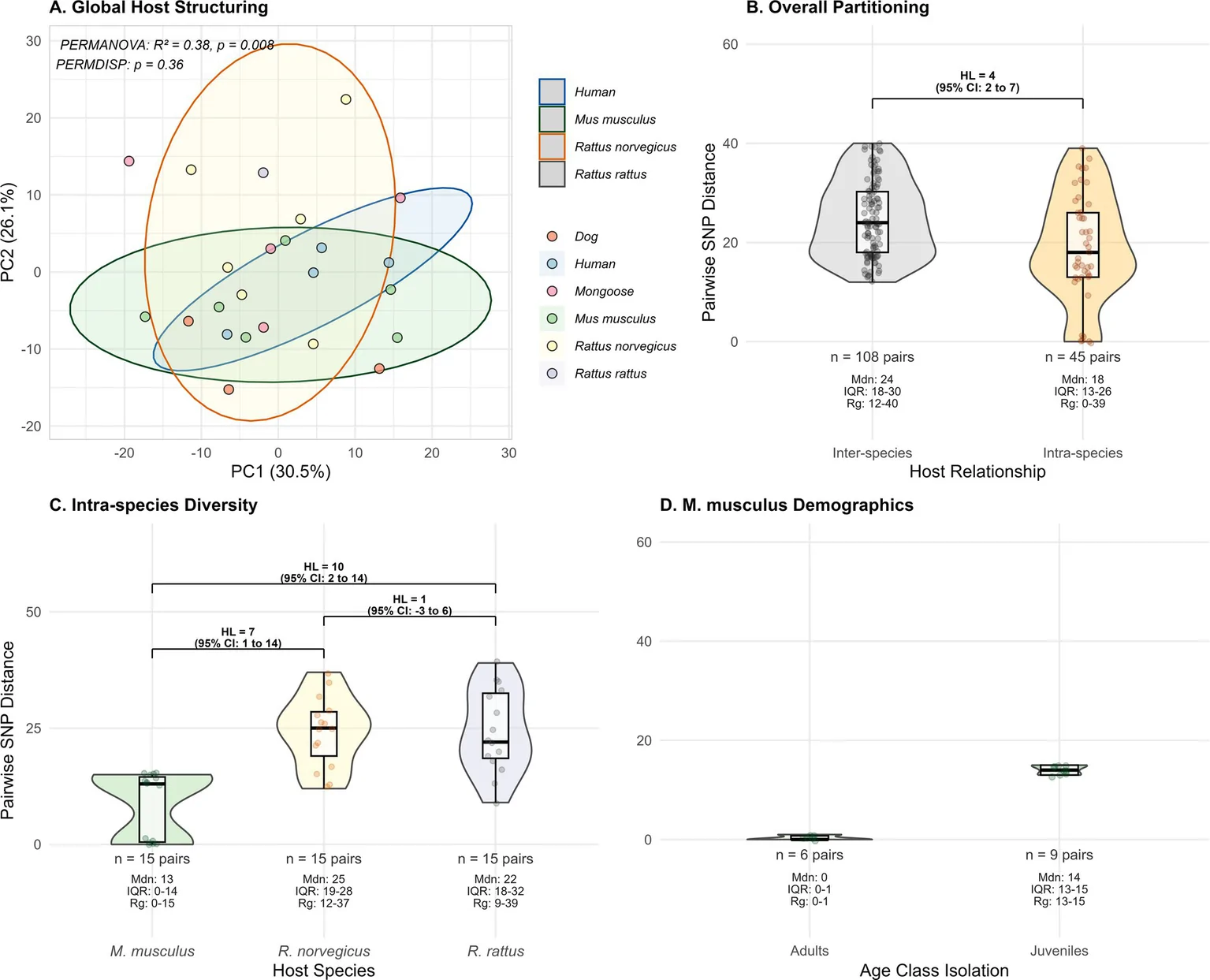
Host-associated genomic structuring and localized demographic variation of *Leptospira interrogans*. **(A)** Principal coordinate analysis (PCoA) of core-genome SNP distances across all sampled host species, with 80% data ellipses shaded by host species. **(B)** Pairwise SNP distance partitioning between inter-species and intra-species host relationships. **(C)** Intra-species core-genome diversity across sympatric rodent reservoirs (*Mus musculus*, *Rattus norvegicus*, and *Rattus rattus*). **(D)** Demographic micro-evolutionary patterns within *M. musculus* transmission chains stratified by age class. Brackets indicate Hodges-Lehmann (HL) median shifts alongside their respective 95% confidence intervals. Internal boxplots show medians and interquartile ranges (IQR); text blocks detail sample size (*n* pairs), median (mdn), IQR, and range (rg).

Host species origin structured *L. interrogans* core-genome SNP diversity, accounting for 38% of the observed variance (PERMANOVA: *R^2^* = 0.38, *F* = 2.19, *p* = 0.008; PERMDISP: *p* = 0.36). PCoA analysis (57% cumulative variance; Figure 5A) showed overlap among the host groups; though host origin affects variance partitioning, the individual isolates do not resolve into completely discrete groupings. When subsetted to rodents, genomic diversity was structured by specific rodent species (PERMANOVA: *R^2^* = 0.34; *F* = 4.32; *p* = 0.002) with homogeneous group dispersions (PERMDISP: *p* = 0.44). Overall, intra-species pairs were more related than inter-species pairs (HL shift = 4 SNPs; 95% CI: 2–7; Figure 5B). *M. musculus* isolates were conserved and less diverse than those from *R. norvegicus* (HL shift = 7 SNPs; 95% CI: 1–14) and *R. rattus* (HL shift = 10 SNPs; 95% CI: 2–14; Figure 5C). The SNP distances within *M. musculus* showed a bimodal distribution (Figure 5C) linked to host demographics (Figure 5D). Pairs comprising adult mice were near-identical, whereas juvenile mice displayed higher baseline variation (HL shift = 14 SNPs; 95% CI: 13–15; *p* = 0.001).

### 3.4 *Leptospira borgpetersenii* genomic results

#### 3.4.1 Core genomic profile and geographic structuring

**Figure 6:**
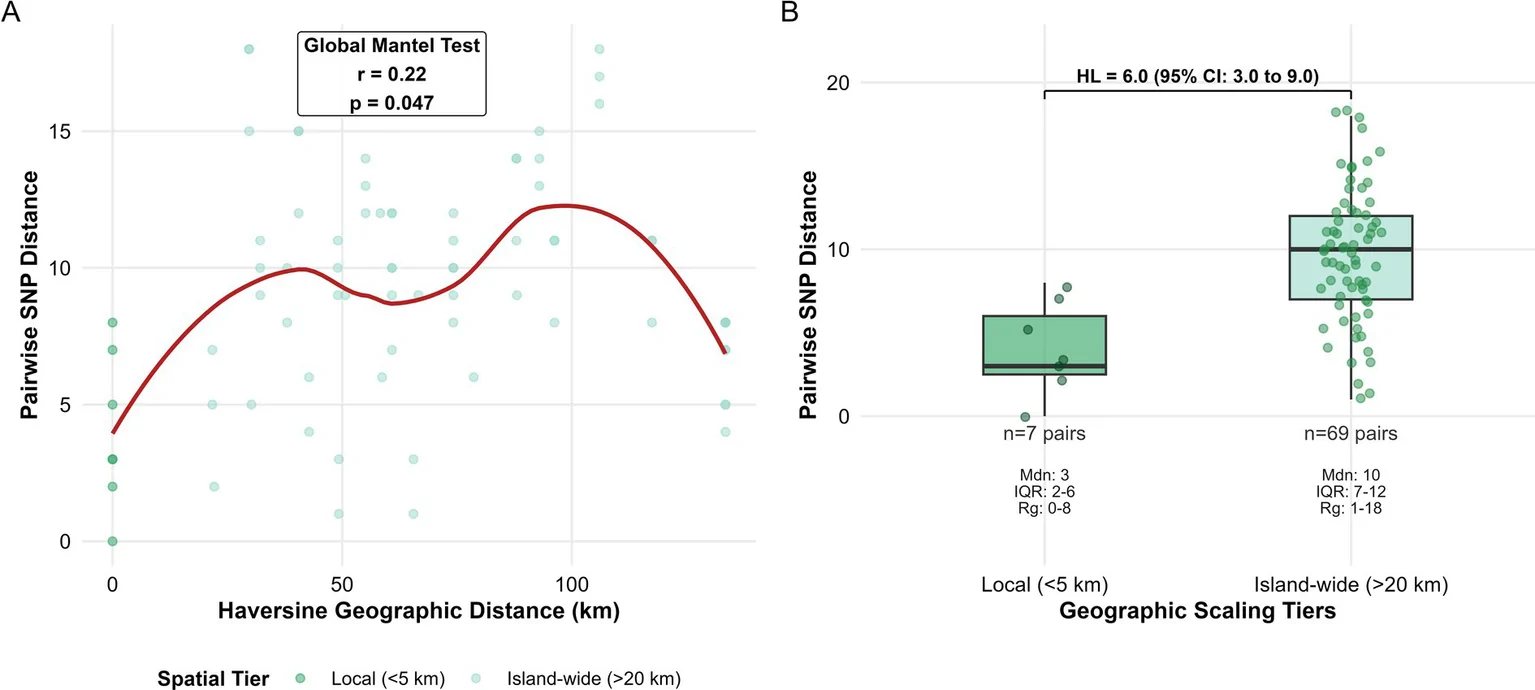
Binary spatial partitioning and micro-geographic structuring of *Leptospira borgpetersenii* core genomes. **(A)** Absolute isolation-by-distance decay plot mapping pairwise core SNP distances directly against continuous Haversine geographic distances (km), overlaid with a local polynomial regression fitting (LOESS) and global Mantel test statistics (*r* = 0.22, *p* = 0.047). Individual data points are color-coded by their corresponding spatial tier. **(B)** Pairwise core-genome SNP distances stratified across two geographic distance tiers: local (<5 km; *n* = 7 pa*irs) an*d island-wide (>20 km; *n* = 69 pairs). Text overlays along the *x*-axis display group-specific descriptive statistics (median [mdn], IQR, and range [rg]). Horizontal brackets indicate the calculated Hodges-Lehmann (HL) median difference effect sizes alongside their respective 95% confidence intervals.

Two bovine isolates were excluded from fine-scale transmission analysis due to divergence (>40,000 SNPs) from the main population. This distance reflects a phylogenomic split separating distinct host-adapted lineages on the island: the excluded cattle-restricted *L. borgpetersenii* serovar Hardjo isolates (16) versus the localized, rodent-specialized serovar Ballum maintenance network evaluated here (14). Retaining these divergent lineages would introduce macro-evolutionary strata that mask local micro-transmission dynamics. The remaining 13 genomes exhibited a median pairwise distance of 9 SNPs (IQR: 7–12, range: 0–18; Supplementary Figure S7). ZCTA boundaries explained 91% of total genomic variance (*F* = 7.24; *p* < 0.001; PERMDISP *p* = 0.33). The Mantel test indicated spatial correlation (r = 0.22, *p* = 0.047; Figure 6A); however, jackknife sensitivity analysis showed this continuous signature was fragile, losing statistical significance (*p* ≥ 0.05) in 8/13 (62%; 95% CI: 32–86) of iterative single-isolate omissions.

Geographic scaling revealed a binary split rather than a progressive decay gradient (Figure 6B). Local pairs (<5 km) had a median of 3 SNPs (IQR: 2.5–6), while island-wide pairs (>20 km) had a median of 10 SNPs (IQR: 7–12), with no regional (5–20 km) pairs. HL contrast confirmed that local isolates were shifted closer by a median of 6 SNPs (95% CI: 3–9). Due to the low overall genomic diversity across the island, the proportion of closely related pairs co-located within the same ZCTA remained low across all three thresholds: at the stringent ≤1 SNP threshold, 1/3 (33%; 95% CI: 6–79) of pairs were co-located within the same ZCTA, remaining stable at 5/16 (31%; 95% CI: 14–55) at ≤5 SNPs, and 10/47 (21%; 95% CI: 11–36) at ≤10 SNPs.

#### 3.4.2 SNP-based genomic clusters and spatiotemporal linkages

**Figure 7:**
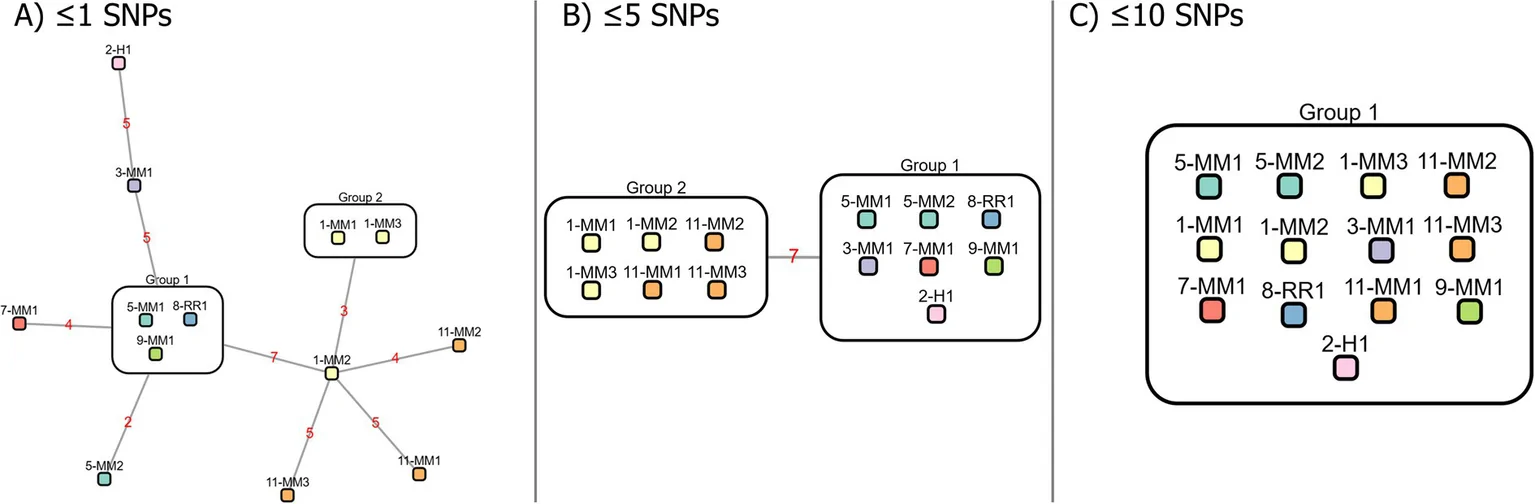
Minimum spanning trees (MSTs) of *Leptospira borgpetersenii* isolates demonstrating genomic clustering behavior across fixed thresholds of **(A)** ≤ 1 SNP, **(B)** ≤ 5, and **(C)** ≤ 10 SNPs. Nodes are color-coded by geographic sampling site and labeled with their unique isolate identifier (denoting sampling site, host species [*Mus musculus* (MM), *Rattus rattus* (RR), human (H)], and isolate number). Edge labels and red numbers indicate absolute pairwise SNP distances between connected nodes.

Fixed genomic thresholds of ≤1, ≤5, and ≤10 SNPs (although similar results were obtained at thresholds down to 7 SNPs) resolved 2, 2, and 1 distinct cluster, respectively (Figure 7; Table 2). At the ≤1-SNP threshold, clusters contained 2 to 3 isolates spanning distinct collection sites and temporal periods, and host species were mixed. Within Group 1, a historical 2015 *M. musculus* anchor clustered in near-identity with an active 2021 *R. rattus* isolate (0–1 SNP divergence) separated by substantial physical distance (Table 2; Supplementary Figure S5).

**Table 2:** Cluster groupings and spatiotemporal and host demographics characteristics of *L. borgpetersenii* isolates at SNP thresholds of ≤1, ≤5, and ≤10.

Cluster ID	Isolate ID	Collection date	Host species	Sex/age	Spatial classification
≤1 SNP Threshold					
Group 1	5-MM1	April 2021	*M. musculus*	Male/Adult	Distant
	8-RR1	March 2021	*R. rattus*	Male/Juvenile	
	9-MM1	2015	*M. musculus*	–	
Group 2	1-MM1	April 2021	*M. musculus*	Male/Juvenile	Same
	1-MM3	April 2021	*M. musculus*	Male/Adult	
≤5 SNP Threshold					
Group 1	5-MM1	April 2021	*M. musculus*	Male/Adult	Distant
	5-MM2	April 2021	*M. musculus*	Male/Adult	
	8-RR1	March 2021	*R. rattus*	Male/Juvenile	
	3-MM1	March 2021	*M. musculus*	Male/Juvenile	
	7-MM1	March 2021	*M. musculus*	Male/Juvenile	
	2-H1	November 2020	Human	–	
	9-MM1	2015	*M. musculus*	–	
Group 2	1-MM1	April 2021	*M. musculus*	Male/Juvenile	Distant
	1-MM2	April 2021	*M. musculus*	Male/Adult	
	1-MM3	April 2021	*M. musculus*	Male/Adult	
	11-MM1	May 2021	*M. musculus*	Female/Adult	
	11-MM2	May 2021	*M. musculus*	Female/Juvenile	
	11-MM3	May 2021	*M. musculus*	Male/Juvenile	
≤10 SNP Threshold					
Group 1	5-MM1	April 2021	*M. musculus*	Male/Adult	Distant
	5-MM2	April 2021	*M. musculus*	Male/Adult	
	1-MM1	April 2021	*M. musculus*	Male/Juvenile	
	1-MM2	April 2021	*M. musculus*	Male/Adult	
	1-MM3	April 2021	*M. musculus*	Male/Adult	
	11-MM1	May 2021	*M. musculus*	Female/Adult	
	11-MM2	May 2021	*M. musculus*	Female/Juvenile	
	11-MM3	May 2021	*M. musculus*	Male/Juvenile	
	7-MM1	March 2021	*M. musculus*	Male/Juvenile	
	2-H1	November 2020	Human	–	
	3-MM1	March 2021	*M. musculus*	Male/Juvenile	
	8-RR1	March 2021	*R. rattus*	Male/Juvenile	
	9-MM1	2015	*M. musculus*	–	

At the ≤5-SNP threshold, Group 1 expanded to include four additional isolates, including three *M. musculus* isolates and a 2020 human clinical case, shifting its spatial classification to a Distant footprint, while Group 2 expanded to include six *M. musculus* isolates spanning two physically distant sites. At the ≤10-SNP threshold, all isolates were contained within a single cluster (Table 2; Figure 7). No clusters showed spatiotemporal linkages except for Group 2 at the ≤1-SNP threshold.

#### 3.4.3 Host-associated genomic homogeneity

Variation across host species reflected a unified population structure. Host species identity accounted for 26% of the core-genome SNP variance among *L. borgpetersenii* isolates, though this association was not statistically significant (PERMANOVA: *F* = 1.75, *p* = 0.13), and dispersion profiles exhibited heterogeneity (PERMDISP: *p* = 0.03). In pairwise comparisons, genomic distances between intra-species pairs were comparable to those of inter-species pairs, exhibiting a median difference shift of 1 SNP (HL shift = 1 SNP; 95% CI: 1–4; Figure 8A). Within the primary reservoir cohort, *M. musculus* isolate pairs (*n* = 55) maintained a baseline median distance of 9 SNPs (IQR: 6–11.5, range: 0–15; Figure 8B). This uniform diversity profile persisted across host species boundaries, confirming an absence of structural segregation between host species.

**Figure 8:**
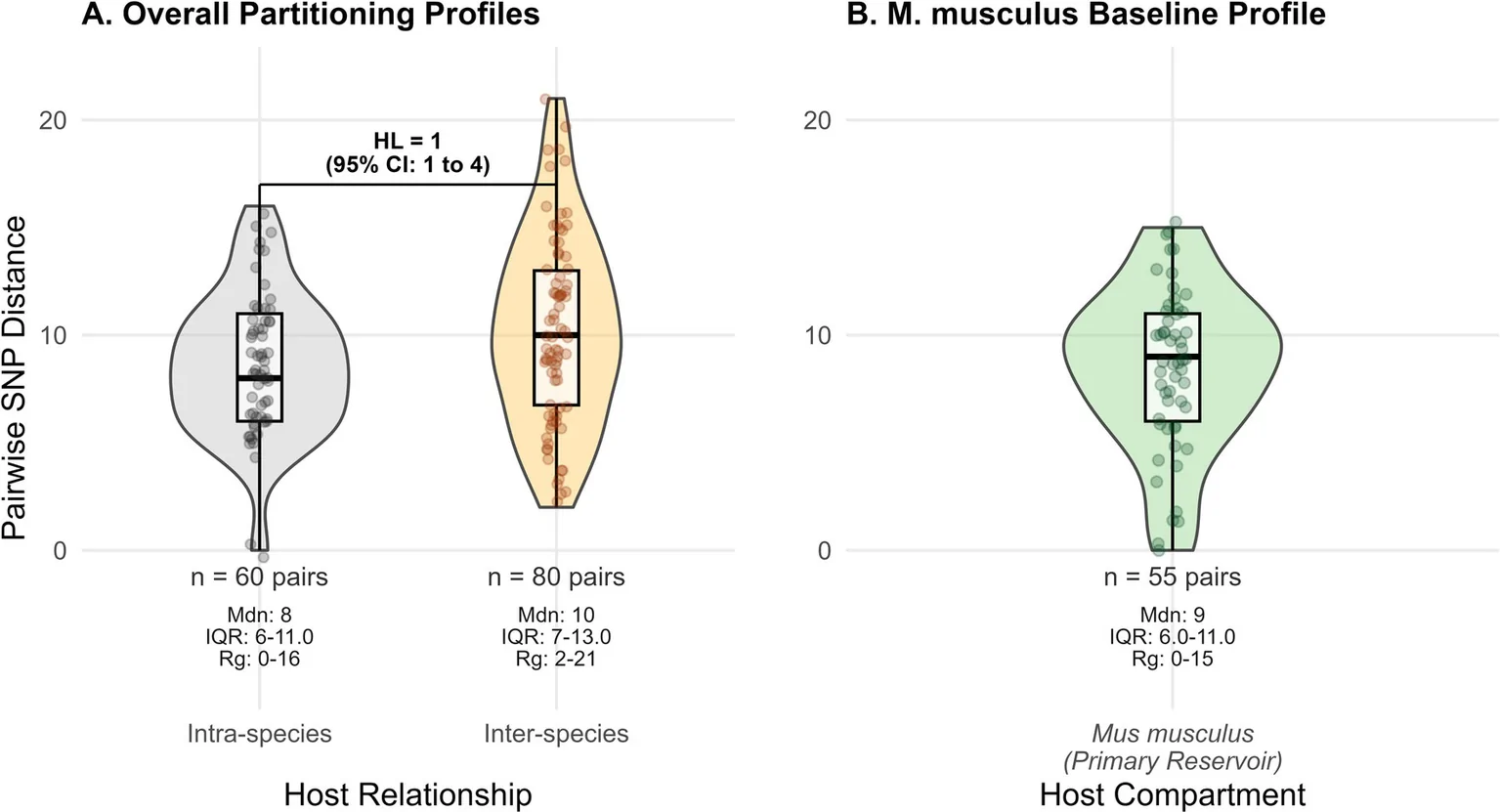
Host-independent genomic homogeneity and reservoir compression of *Leptospira borgpetersenii*. **(A)** Overall, pairwise core-genome SNP partitioning demonstrating overlapping diversity profiles between intra-species and inter-species host relationships. The horizontal bracket displays the Hodges–Lehmann (HL) median shift with 95% confidence intervals. **(B)** Compressed baseline genomic profile within the primary host *Mus musculus*, highlighting a lack of host species partitioning. Internal boxplots illustrate medians and interquartile ranges (IQR); textual overlays display exact pair numbers (*n*), medians (mdn), IQRs, and absolute ranges (rg).

### 3.5 *Leptospira kirschneri* genomic results

Five *L. kirschneri* genomes derived from two host species across four sampling sites were evaluated (Supplementary Figure S2.3). The total population exhibited a median pairwise distance of 23 SNPs (IQR: 21–25, range: 12–28). This core micro-evolutionary landscape is visualized in the population-wide genomic distance matrix (Supplementary Figure S8). Notably, two isolates co-located at site S11—derived from a human case (11-H13; January 2021) and a *Mus musculus* reservoir (11-MM12, May 2021)—differed by 12 SNPs.

## 4 Discussion

This genomic epidemiology study shows how systems thinking—simultaneously evaluating pathogen micro-evolution, host variability, and structured landscape connectivity—is essential for decoding endemic *Leptospira* population dynamics. Rather than treating leptospirosis as a uniform public health threat, a systems-level approach reveals that co-circulating *Leptospira* species occupy distinct evolutionary and ecological niches across host-environment interfaces.

### 4.1 Comparative root-to-tip temporal dynamics and evolutionary niches

Stratified root-to-tip regression analysis showed that a large sample volume is not a prerequisite for capturing robust micro-evolutionary trends in endemic settings. By evaluating target species independently, estimation biases that occur when pooling diverse lineages with asymmetric evolutionary rates are circumvented (30). This approach identified a biological divergence where *L. interrogans* maintains an active, contemporary molecular clock (*R*^2^ = 0.30), indicating ongoing mutation, whereas *L. borgpetersenii* exhibits temporal genomic homogeneity (*R*^2^ = 0.00).

The complete collapse of the *L. borgpetersenii* temporal signal upon removing its 2015 historical anchor shows the necessity of micro-timeline sensitivity sub-runs. Without this step, standard molecular clock models suffer from leverage artifacts where a single historical isolate forces a false-positive linear evolutionary rate across a population that lacks a measurable contemporary temporal signal (22, 31). This lack of short-term temporal structure likely indicates that the 2021 sampling window contains insufficient temporal resolution for time-calibrated phylogenomic inference. While *L. interrogans* accumulates detectable core-genome variation within this timeframe, tracking the evolutionary trajectory of *L. borgpetersenii* requires expanded temporal depth beyond contemporary sampling baselines to confidently separate true sequence conservation from a simple lack of phylogenomic signal.

### 4.2 Landscape connectivity and spatial decay

Geographic landscape features structure pathogenic *Leptospira* lineages in a species-specific manner. ZCTA boundaries account for 56% of core-genomic variance in *L. interrogans* compared to 91% in *L. borgpetersenii* (PERMANOVA). Although small sample sizes within individual administrative units necessitate conservative interpretation, this contrast in variance partitioning likely reflects distinct spatial structures: a highly connected population for *L. interrogans* versus a fragmented, geographically subdivided population for *L. borgpetersenii* (27).

*L. interrogans*: This species maintains high landscape connectivity island-wide, exhibiting a median divergence of 24 SNPs at distances exceeding 20 km. This suggests that urban infrastructure and human-mediated transport networks act as active conduits for long-distance dispersal. Moreover, at the stringent ≤1 SNP threshold, geographic tracking fidelity is absolute (100% co-located). Low SNP distances across space alone could imply a static, slowly evolving population that undergoes minimal change over time. However, when paired with the root-to-tip analysis, this localized spatial clustering provides a potential signal of recent transmission. Because *L. interrogans* maintains an active contemporary molecular clock (*R^2^* = 0.30), the combination of ongoing mutation and absolute geographic co-location indicates that these near-identical genomic matches likely represent point-source exposures and active transmission networks rather than shared ancestral background variation (10).

*L. borgpetersenii*: This species displays a narrow baseline of genomic variation rather than a progressive spatial decay gradient. Local pairs remain highly uniform (median: 3 SNPs), while island-wide pairs baseline at a median of 10 SNPs. When interpreted alongside the root-to-tip sensitivity analysis (*R^2^* = 0.00), this minimal genomic distance likely reflects high core-genome conservation over short evolutionary timelines rather than active physical containment. This temporal genomic homogeneity explains why, at the stringent ≤1-SNP threshold, only 33% of pairs are co-located. Because the lineage accumulates mutations slowly within its primary host niche, near-identical clones persist across geographically distant regions of the island. This pattern likely points to a historical mass dispersion event followed by limited subsequent mutation, leaving a uniform background population maintained by *M. musculus*. Alternatively, it could mean that sampling missed the true primary host or source on the island, with these mice representing separate spillover events from an uncaptured reservoir.

### 4.3 Calibration of fixed transmission clusters

Applying uniform genomic thresholds across the genus *Leptospira* would introduce misclassification. For *L. interrogans*, a relaxed threshold of ≤10 SNPs successfully captures localized, spatiotemporally linked networks without losing resolution. For *L. borgpetersenii*, however, the entire population groups into a single undifferentiated cluster at ≤10 SNPs, likely masking active transmission dynamics. This structural difference is a consequence of distinct genomic architectures and ecological survival strategies:

*Environmental restriction and serovar-level host specialization:* Comparative genomic analysis by Bulach et al. (32) showed that *L. borgpetersenii* undergoes insertion-sequence-mediated genome reduction, losing genes tied to environmental sensing and nutrient transport. Because these lost pathways restrict the pathogen’s metabolic viability outside a host organism, this genomic reduction favors an evolutionary reliance on direct host-to-host transmission cycles. While this evolutionary shift was originally characterized in cattle-restricted serovar Hardjo isolates (32), the empirical findings indicate that these host-dependence constraints similarly influence transmission within local rodent populations. The macro-evolutionary divergence (>40,000 SNPs) separating bovine-isolated serovar Hardjo lineages (16) from the rodent-isolated serovar Ballum network (14) likely supports a model of serovar-host specificity. Driven by historical genome reduction, these sub-populations likely follow independent pathways toward increased maintenance-host-reliance, sustaining the pathogen within ecologically partitioned biological reservoirs. However, because these observations rest on a global genome-wide distance framework, they represent a hypothesized evolutionary model; gene-by-gene selection pressure analyses are required to separate cellular stasis from targeted selective sweeps.

*Environmental stability and multi-host geographic dispersal:* In contrast to the genomic reduction observed in *L. borgpetersenii*, *L. interrogans* retains a large, structurally intact genome that includes genes optimized for survival in soil and water (32). This genomic structure supports persistence outside the host, allowing surface water and soil matrices to function as shared vehicles for transmission across the landscape. Because aquatic and soil environments preserve shed *L. interrogans* rather than acting as immediate dead ends, environmental runoff or shared habitats can facilitate the indirect spread of closely related isolates among different hosts. This mechanism is consistent with the cluster analysis in Section 3.3.2, where *L. interrogans* displayed multi-host sharing—evidenced by closely related matches bridging humans, dog, rodents, and mongoose—alongside a broad geographic distribution across collection sites. Furthermore, the finding that isolates from the same sites were not always subsumed by a single genomic cluster indicates that high genomic diversity can exist even at highly localized positions. This genomic signature aligns with environmental baseline data from Puerto Rico (15), where *L. interrogans* lineages were widespread throughout local soil and surface water channels, supporting a model where environmental stability contributes to wide-area pathogen dispersal.

### 4.4 Host species and demographics

Rodent population structures appear to be associated with *Leptospira* genomic partitioning. For *L. interrogans*, host species identity accounts for 38% of total core-genome variance, resolving into distinct signatures among sympatric *R. norvegicus*, *R. rattus*, and *M. musculus* populations. Conversely, *L. borgpetersenii* shows an absence of host-species partitioning (*R*^2^ = 0.26, *p* = 0.13), operating as a shared pool dominated by *M. musculus*. Within these networks, bacterial genomic relationships mirror host behavioral ecology:

*Zoonotic spillover paths:* The close genomic association of *L. interrogans* from a human case and *R. norvegicus* across a two-month interval suggests a reservoir sustained by local rodent populations. When tracking thresholds expand to ≤10 SNPs, this localized chain broadens into a contiguous multi-species network encompassing a mongoose isolate and additional sympatric *R. norvegicus* (Table 1; Figure 4C). This integration highlights potential cross-species spillover sharing transmission pathways. Conversely, the *L. borgpetersenii* human case does not resolve into a localized point-source network (Table 2). The human isolate only links at a relaxed ≤5 SNP threshold, clustering within an expanded cohort of *M. musculus* hosts spanning physically distant sites. This lack of localized spatiotemporal tracking, combined with widespread genomic homogeneity that bypasses individual site boundaries, suggests the human acquired the infection from a widely dispersed, island-wide background clonal lineage rather than a recent, point-source transmission chain.

*Transmission patterns by rodent age and sex:* Genomic data links directly to specific behaviors across different rodent groups (Supplementary Figure S6). In *L. interrogans*, the tight clustering of isolates from male and female adult pairs collected concurrently (genomic Groups 1, 2, and 4; Table 1) aligns with potential sexual or reproductive transmission routes reported in rodents (4, 33). Furthermore, *L. interrogans* isolates within *M. musculus* display a distinct bimodal SNP distribution linked to demographics (Figure 5D). Pairs comprising adult mice were near-identical, whereas transmission chains involving juvenile mice displayed higher genomic variation. This demographic contrast is supported by a Hodges–Lehmann median shift of 14 SNPs, suggesting age-dependent environmental exposure, differential shedding kinetics, or rapid juvenile dispersal before stable endemic infection sets in. Within *L. borgpetersenii M. musculus* networks, the clustering of juvenile and adult female isolates at the ≤5 SNP threshold points to potential vertical or maternal-behavioral transmission pathways, such as nest sharing, grooming, or suckling (Table 1; Supplementary Figure S6) (33).

### 4.5 *Leptospira kirschneri* distribution dynamics

While the small sample size prevented the calculation of species-specific transmission thresholds, *L. kirschneri* exhibited the highest overall genomic baseline diversity. A multi-species link was detected in which a human and *M. musculus* isolate from the same site diverged by 12 SNPs across a four-month window. Given the relatively high SNP distance as compared to the other two *Leptospira* species and the fact that *L. kirschneri* represents a minor fraction of the current dataset, these rodents likely represent incidental hosts or indicators of a shared, unsampled primary reservoir rather than components of a direct transmission chain. This genomic gap highlights that an important part of the local *L. kirschneri* reservoir remains uncharacterized, demonstrating the utility of core-genome alignments to identify gaps in sampling within endemic tropical environments.

### 4.6 Temporal persistence and public health implications

Cross-cohort genomic matches spanning multiple years, such as a 2018 dog isolate linking to a 2021 *R. norvegicus* isolate, indicate that these pathogenic lineages are stable and persistent. Rather than being driven by repeated external introductions, data suggests that endemic *Leptospira* is likely sustained by conserved localized reservoirs. To apply these insights, spatial tracking data should be interpreted alongside evolutionary rates to effectively guide field epidemiology. For *L. interrogans*, where an active molecular clock rapidly differentiates lineages over time, site-focused reactive sampling is highly precise for mapping active transmission chains. Conversely, tracking *L. borgpetersenii* requires a broader, proactive geographic footprint; because genomic homogeneity causes nearly identical sequences to persist across distant regions, localized sampling alone cannot reliably differentiate a fresh local outbreak from baseline, island-wide background uniformity. These distinct spatial and temporal patterns carry practical implications for public health molecular surveillance and for control strategies.

### 4.7 Data limitations

This study relies on opportunistic sequence data, which introduces geographic and host sampling gaps. However, the repeated detection of identical *Leptospira* lineages within targeted host niches indicates that convenience sampling identifies primary pathogen-host relationships driving endemic persistence. Future surveillance frameworks should prioritize denser longitudinal sampling and concurrent environmental WGS (soil and water) to differentiate active transmission chains from shared environmental exposures.

Methodologically, while the non-independence of pairwise matrices limits standard confidence intervals, the use of post-hoc bootstraps (Supplementary methods) and jackknife analyses ensures that the structural findings remain statistically meaningful. Given that species-specific *in vivo* mutation rates and exact cellular generation times remain poorly defined across the genus *Leptospira*, integrating forward-time evolutionary simulations will be a necessary next step. Furthermore, executing formal locus-specific recombination mapping and gene-by-gene selection pressure analyses will be necessary in future investigations to cleanly partition true genomic homogeneity from background homologous recombination and selective sweeps within these reservoir populations.

## 5 Conclusion

This study shows that co-circulating pathogenic *Leptospira* species occupy distinct ecological and evolutionary niches in Puerto Rico. *L. interrogans* transmission is shaped by high environmental persistence, structural landscape connectivity, and clear host-species partitioning. Conversely, *L. borgpetersenii* relies on a highly uniform, host-adapted clonal pool characterized by short-term genomic homogeneity within small mammal populations. Genomic surveillance frameworks cannot rely on a single, universal genus-wide transmission threshold; they must be ecologically stratified and species-specific to accurately map spatiotemporal spread and mitigate zoonotic risk.

## Data Availability

The original contributions presented in the study are included in the article/Supplementary material, further inquiries can be directed to the corresponding author.
