# Supplementary material for: Genomic and ecological systems-thinking framework for pathogenic Leptospira in Puerto Rico
Source: Front Public Health. 2026 Aug 31;14:1838089. doi: 10.3389/fpubh.2026.1838089 (PMC13572634; doi:10.3389/fpubh.2026.1838089)
Supplement: Supplementary file 1 [file Supplementary_file_1.DOCX]

***Supplementary Material***

# 1 Supplementary Data

**Table S1.** The sixty-seven publicly available *Leptospira* whole-genome sequences and metagenomes from Puerto Rico (2015 to 2021) were initially evaluated in this study.

| Run | BioSample | Organism | Collection Date |
| --- | --- | --- | --- |
| BioProject: PRJNA766613 | | | |
| SRR16134453 | SAMN21855215 | *Leptospira sanjuanensis* | 2/13/2020 |
| SRR16134454 | SAMN21855214 | *Leptospira sanjuanensis* | 2/13/2020 |
| SRR16134455 | SAMN21855213 | *Leptospira bandrabouensis* | 2/13/2020 |
| SRR16134456 | SAMN21855212 | *Leptospira bandrabouensis* | 2/13/2020 |
| SRR16134457 | SAMN21855211 | *Leptospira bandrabouensis* | 2/13/2020 |
| SRR16134458 | SAMN21855210 | *Leptospira bandrabouensis* | 2/13/2020 |
| SRR16134459 | SAMN21855209 | *Leptospira mtsangambouensis* | 2/13/2020 |
| SRR16134460 | SAMN21855208 | *Leptospira levettii* | 2/13/2020 |
| SRR17853364 | SAMN21855215 | *Leptospira sanjuanensis* | 2020 |
| SRR17853365 | SAMN21855214 | *Leptospira sanjuanensis* | 2020 |
| BioProject: PRJNA937758 | | | |
| SRR23749863 | SAMN33419161 | Urine metagenome | 2020 |
| SRR23749864 | SAMN33419161 | Urine metagenome | 2020 |
| SRR23749865 | SAMN33419160 | Urine metagenome | 2020 |
| SRR23749866 | SAMN33419171 | Urine metagenome | 2020 |
| SRR23749867 | SAMN33419171 | Urine metagenome | 2020 |
| SRR23749868 | SAMN33419170 | Blood metagenome | 2020 |
| SRR23749869 | SAMN33419160 | Urine metagenome | 2020 |
| SRR23749870 | SAMN33419170 | Blood metagenome | 2020 |
| SRR23749877 | SAMN33419166 | Soil metagenome | 2020 |
| SRR23749880 | SAMN33419159 | Urine metagenome | 2020 |
| SRR23749881 | SAMN33419159 | Urine metagenome | 2020 |
| BioProject: PRJNA794843 | | | |
| SRR17454385 | SAMN24659832 | *Leptospira borgpetersenii* | 9/9/2021 |
| SRR17454386 | SAMN24659831 | *Leptospira borgpetersenii* | 3/10/2021 |
| SRR17454387 | SAMN24659830 | *Leptospira santarosai* | 3/26/2021 |
| BioProject: PRJNA1111116 | | | |
| SRR31182823 | SAMN44490563 | *Leptospira borgpetersenii* | 2021 |
| SRR31182824 | SAMN44490562 | *Leptospira borgpetersenii* | 2021 |
| SRR31182825 | SAMN44490561 | *Leptospira borgpetersenii* | 2021 |
| SRR31182826 | SAMN44490560 | *Leptospira borgpetersenii* | 2021 |
| SRR31182827 | SAMN44490559 | *Leptospira borgpetersenii* | 2021 |
| SRR31182828 | SAMN44490596 | *Leptospira kirschneri* | 2021 |
| SRR31182829 | SAMN44490595 | *Leptospira kirschneri* | 2021 |
| SRR31182830 | SAMN44490594 | *Leptospira kirschneri* | 2021 |
| SRR31182831 | SAMN44490558 | *Leptospira borgpetersenii* | 2021 |
| SRR31182832 | SAMN44490593 | *Leptospira kirschneri* | 2021 |
| SRR31182833 | SAMN44490592 | *Leptospira kirschneri* | 1/28/2021 |
| SRR31182834 | SAMN44490591 | *Leptospira interrogans* | 2021 |
| SRR31182835 | SAMN44490590 | *Leptospira interrogans* | 2021 |
| SRR31182836 | SAMN44490589 | *Leptospira interrogans* | 2021 |
| SRR31182837 | SAMN44490588 | *Leptospira interrogans* | 2021 |
| SRR31182838 | SAMN44490587 | *Leptospira interrogans* | 2021 |
| SRR31182839 | SAMN44490586 | *Leptospira interrogans* | 2021 |
| SRR31182840 | SAMN44490585 | *Leptospira interrogans* | 2021 |
| SRR31182841 | SAMN44490584 | *Leptospira interrogans* | 2021 |
| SRR31182842 | SAMN44490557 | *Leptospira borgpetersenii* | 2021 |
| SRR31182843 | SAMN44490583 | *Leptospira interrogans* | 2021 |
| SRR31182844 | SAMN44490582 | *Leptospira interrogans* | 2021 |
| SRR31182845 | SAMN44490581 | *Leptospira interrogans* | 2021 |
| SRR31182846 | SAMN44490580 | *Leptospira interrogans* | 2021 |
| SRR31182847 | SAMN44490579 | *Leptospira interrogans* | 2021 |
| SRR31182848 | SAMN44490578 | *Leptospira interrogans* | 2021 |
| SRR31182849 | SAMN44490577 | *Leptospira interrogans* | 2021 |
| SRR31182850 | SAMN44490576 | *Leptospira interrogans* | 2021 |
| SRR31182851 | SAMN44490575 | *Leptospira interrogans* | 2021 |
| SRR31182852 | SAMN44490574 | *Leptospira interrogans* | 2021 |
| SRR31182853 | SAMN44490556 | *Leptospira borgpetersenii* | 2021 |
| SRR31182854 | SAMN44490573 | *Leptospira interrogans* | 2021 |
| SRR31182855 | SAMN44490572 | *Leptospira interrogans* | 2021 |
| SRR31182856 | SAMN44490571 | *Leptospira interrogans* | 2021 |
| SRR31182857 | SAMN44490570 | *Leptospira interrogans* | 2018 |
| SRR31182858 | SAMN44490569 | *Leptospira interrogans* | 3/3/2021 |
| SRR31182859 | SAMN44490568 | *Leptospira interrogans* | 2/18/2021 |
| SRR31182860 | SAMN44490567 | *Leptospira borgpetersenii* | 2021 |
| SRR31182861 | SAMN44490566 | *Leptospira borgpetersenii* | 2021 |
| SRR31182862 | SAMN44490565 | *Leptospira borgpetersenii* | 2021 |
| SRR31182863 | SAMN44490564 | *Leptospira borgpetersenii* | 2021 |
| SRR31182864 | SAMN44490554 | *Leptospira borgpetersenii* | 2015 |
| SRR31182865 | SAMN44490553 | *Leptospira borgpetersenii* | 11/27/2020 |

 **Figure S1.** Distance-based dendrogram of 53 *Leptospira* genomes from Puerto Rico (2015 to 2021) inferred using whole genome sequences Mash distances. Tip labels represent host species, while tip colors indicate *Leptospira* species. The dendrogram is rooted with *L. mtsangambouensis* as the outgroup. Clustering aligns with established *Leptospira* taxonomy, delineating the pathogenic P1^+^ clade (*L. interrogans* [n=24], *L. kirschneri* [n=5]*,* *L. borgpetersenii* [n=15], and *L. santarosai* [n=1]); the pathogenic P1^-^ clade (*L. sanjuanensis* [n=2]); and saprophytic S1 clade (*L. bandrabouensis* [n=4], *L. levettii* [n=1], and the *L. mtsangambouensis* outgroup [n=1]).

**Table S2.** Parameters and default configuration options used for *Leptospira* species whole genomic sequences analysis within Bactopia v3.1.0.

| Parameter | Description |
| --- | --- |
| Snippy Parameters | |
| --mapqual | Minimum read mapping quality to consider Type: integer, Default: 60 |
| --basequal | Minimum base quality to consider Type: integer, Default: 13 |
| --mincov | Minimum site depth to for calling alleles Type: integer, Default: 10 |
| --minfrac | Minimum proportion for variant evidence (0=AUTO), Type: integer |
| --minqual | Minimum QUALITY in VCF column 6 Type: integer, Default: 100 |
| --maxsoft | Maximum soft clipping to allow Type: integer, Default: 10 |
| Snippy-Core Parameters | |
| --maxhap | Largest haplotype to decompose Type: integer, Default: 100 |
| --mask | BED file of sites to mask Type: string |
| --mask_char | Masking character Type: string, Default: X |
| Gubbins Parameters | |
| --iterations | Maximum number of iterations Type: integer, Default: 5 |
| --min_snps | Min SNPs to identify a recombination block Type: integer, Default: 3 |
| --min_window_size | Minimum window size Type: integer, Default: 100 |
| --max_window_size | Maximum window size Type: integer, Default: 10000 |
| --filter_percentage | Filter out taxa with more than this percentage of gaps Type: number, Default: 25 |

**Figure S2.** Maximum-likelihood phylogenies and spatial relationships of *Leptospira* isolates. Maximum-likelihood phylogenetic trees were visualized in R v4.3.1 using phytools v2.3.0, linking tree nodes to sampling sites to explore evolutionary relationships and geographic structuring of *L. interrogans* (n=24), *L. borgpetersenii* (n=13), and *L. kirschneri* (n=5) isolates collected in Puerto Rico from 2015 to 2021. The best-fit substitution model (K2P) was selected using the Bayesian information criterion (BIC) via ModelFinder, and node support was assessed with 1,000 bootstrap replicates. Bootstrap values >60% are indicated by light blue circles. Each node represents an isolate sequence and is connected to its collection site in Puerto Rico by a dotted line. Node colors represent sampling sites.

**Figure S2.1 (*L. interrogans*).** The maximum likelihood tree demonstrates that certain *L. interrogans* isolates were closely related to one another, often corresponding to the same sampling sites or host species. For instance, close relationships were observed among isolates from *M. musculus* at site S10, *R. rattus* at site S11, three isolates from site S2 (2-RR2, 2-RR3, 2-RR4), and two from site S3 (3-RR2, 3-RR3). Isolates from rodents and other host species also appeared in close phylogenetic proximity, such as an isolate from a dog (4-D1) and a *R. norvegicus* (5-RN4), and a mongoose (12-M3) with a *R. norvegicus* (12-RN5). Additionally, isolates from site S11, including a human and three isolates from *R. norvegicus*, were closely related.

**Figure S2.2 (*L. borgpetersenii*).** Site-based spatial relationships were highly pronounced in the *L. borgpetersenii* phylogeny. The maximum likelihood tree revealed that isolates from the same sites or host species were very closely related. For example, three isolates from *M. musculus* at site S11 and three from site S1 showed high relatedness. Isolates from different hosts also grouped closely together, such as an isolate from a human and a *M. musculus* (2-H1 and 3-MM1).

**Figure S2.3 (*L. kirschneri*).** Maximum-likelihood phylogenetic tree (left) and Minimum Spanning Tree (MST) (right) of *L. kirschneri* isolates, demonstrating genomic clusters based on a SNP threshold of ≤12.

**Table S3.** Host demographic profiles and genomic Summaries for retained isolates.

| Host Species | Total Isolates (n) | *Leptospira* Species Assignment (n) | Sex (Male / Female / Unspecified; n) | Age (Adult / Juvenile / Unspecified; n) |
| --- | --- | --- | --- | --- |
| *Mus musculus* (House mouse) | 19 | *L. interrogans* (4)    *L. borgpetersenii* (11)    *L. kirschneri* (4) | 10 / 8 / 1 | 10 / 9 / 0 |
| *Rattus rattus* (Roof rat) | 11 | *L. interrogans* (10)    *L. borgpetersenii* (1) | 5 / 6 / 0 | 10 / 1 / 0 |
| *Rattus norvegicus* (Norway rat) | 7 | *L. interrogans* (7) | 5 / 2 / 0 | 7 / 0 / 0 |
| *Herpestes auropunctatus* (Mongoose) | 1 | *L. interrogans* (1) | 1 / 0 / 0 | 1 / 0 / 0 |
| *Canis lupus familiaris* (Domestic dog) | 1 | *L. interrogans* (1) | 0 / 0 / 1 | 0 / 0 / 1 |
| *Homo sapiens* (Human) | 4 | *L. interrogans* (1)    *L. borgpetersenii* (1)    *L. kirschneri* (2) | 4 / 0 / 0 | 4 / 0 / 0 |
| Total Population | 44 | - | 26 / 16 / 2 | 33 / 10 / 1 |

**Figure S3.** Root-to-tip regression analyses demonstrating distinct temporal signals and micro-evolutionary trajectories among co-circulating *Leptospira* species. These visualizations are direct graphical outputs from the Clockor2 v1.10.2 software. Each figure is divided into three main components: (1) a phylogenetic tree on the left, (2) the corresponding root-to-tip regression plot on the right, and (3) a summary statistics table below. For the regression graphs, the x-axis represents the sampling date (time), and the y-axis represents the root-to-tip genetic divergence (distance from the root). The embedded tables display the optimal metrics calculated by Clockor2, including the best-fitting root position, correlation coefficient ($R^{2}$), Akaike Information Criterion corrected for small sample size (AICc), the applied clock model, the number of tips (isolates), the estimated substitution rate, the residual median, and the y-intercept. To differentiate between long-term evolutionary trends and acute dynamics, regressions were evaluated on both macro-timelines (including deep historical anchor isolates) and contemporary micro-timelines (restricted to recent isolates).

**Figure S3.1** *L. interrogans* (macro-timeline): Clockor2 output including both contemporary and historical isolates, demonstrating a baseline linear temporal signal.

**Figure S3.2** *L. interrogans* (micro-timeline): Clockor2 output restricted to contemporary isolates, reflecting an accelerated substitution rate indicative of active host-to-host transmission chains.

**Figure S3.3** *L. borgpetersenii* (macro-timeline): Clockor2 output including historical anchor isolate to provide a long-term temporal baseline.

**Figure S3.4** *L. borgpetersenii* (micro-timeline): Clockor2 output restricted strictly to contemporary isolates. The flattened regression line ($R^{2}$ = 0.000) illustrates extreme genomic stasis over the short-term sampling window.

**Figure S3.5** *L. kirschneri* (contemporary isolates): Clockor2 output capturing the distinct temporal signal and substitution rate indicative of the tight localized transmission.

**Figure S4.** Hierarchical clustering heatmap of pairwise *L. interrogans* core-genome SNP distances. Symmetric matrix illustrating absolute pairwise SNP differences across 24 isolates, transitioning from bright yellow (0 SNPs) to deep violet (35+ SNPs). Top annotation tracks denote host species and landscape classifications. Geographic coordinates were classified using the 2020 USDA Economic Research Service (ERS) primary Rural-Urban Commuting Area (RUCA) codes to define three operational zones: metropolitan: urban centers and primary commuting ties; micropolitan: large towns with intermediate commuting intensity; rural: low-density areas with minimal urban commuting ties.

**Figure S5.** Spatiotemporal timeline schematic and multi-year visualization of *Leptospira* transmission networks. Horizontal trajectories track individual genomic transmission networks across a multi-year spatiotemporal sampling horizon (2015-2021) partitioned by genetic distance and localized capture dynamics. The top panel maps *L. interrogans* networks, highlighting cross-species cohort linkages geographical mixing across time (e.g., the 2018 domestic dog anchor tracking across a wide physical distance 2021 rodent isolates). The bottom panel maps *L. borgpetersenii* networks, illustrating long-term clonal stasis and deep geographic persistence centered within highly conserved localized host reservoirs. Nodes are color-coded by host compartment/category, and dashed baseline lines denote continuous spatiotemporal tracking.

**Figure S6.** Pairwise core-genome SNP distance distributions stratified by host species, sex, and age cohorts. This multi-panel grid displays absolute single nucleotide polymorphism (SNP) variations among intra-species demographic groups for (A) *Leptospira interrogans* and (B) *Leptospira borgpetersenii*. Panels segment the populations by rodent host species (*Mus musculus*, *Rattus norvegicus*, and *Rattus rattus*) and host sex combinations (Female-Female, Male-Female, and Male-Male pairs). Individual x-axis further map intra-pair age-class combinations (Adult-Adult, Adult-Juvenile, and Juvenile-Juvenile). Violin plots display the overall density distributions, internal white box plots represent core medians and interquartile ranges (IQR), and individual jittered points capture exact pairwise genetic distances. Text overlays denote the median (Mdn) SNP value and total pair count (n) within each discrete demographic tier.

**Figure S7.** Hierarchical clustering heatmap of pairwise *L. borgpetersenii* core-genome SNP distances. Symmetric matrix illustrates absolute pairwise SNP differences across 13 localized isolates, transitioning from bright yellow (0 SNPs) to deep violet (15+ SNPs). Top annotation tracks denote host species and landscape classifications. Geographic coordinates were classified using the 2020 USDA Economic Research Service (ERS) primary Rural-Urban Commuting Area (RUCA) codes to define three operational zones: metropolitan: urban centers and primary commuting ties; micropolitan: large towns with intermediate commuting intensity; rural: low-density areas with minimal urban commuting ties.

**Figure S8**. Pairwise genetic distance matrix of *L. kirschneri* core genomes. Symmetric tiling matrix displaying absolute pairwise single nucleotide polymorphism (SNP) differences across five localized isolates. Individual cell values denote the exact core-genome SNP differences between isolate intersections, transitioning across a progressive color gradient from bright cream/yellow (0 SNPs, absolute sequence identity) through cyan (10-15 SNPs) to deep indigo/violet (20+ SNPs, maximum divergence within the cohort). Axis text strings identify individual sequence coordinates labeled by sampling site, host species compartment (*M. musculus* (MM) or *H. sapiens* (H), and unique isolate enumeration tags. The asymmetrical light-cyan tile highlights the minimum baseline divergence (12 SNPs) bridging the human clinical case (11-H13) directly to a localized small mammal reservoir (11-MM12) co-sampled at site S11.

**Supplementary Methods**

**Sensitivity analysis: per-isolate spatial effect size**

To address the statistical non-independence of pairwise SNP distances (where each individual isolate contributes to multiple pairs, creating pseudo replication), we conducted a sensitivity analysis using per-isolate summaries as independent units. For each focal isolate $i$, we computed two metrices: the median SNP distance from isolate $i$ to all other isolates sampled within the same ZCTA $\left( D_{local} \right),$and the median SNP distance from isolate $i$ to isolates sampled from non-contiguous ZCTAs $\left( D_{distant} \right)$. We defined the isolate-level spatial shift as$\Delta_{spatial}=D_{distant}-D_{local}$. This value represents how many SNPs farther an isolate is from non-contiguous locations relative to its own localized ZCTA. We summarized the overall spatial effect size as the median of $\Delta_{spatial}$ across all isolates and estimated uncertainty via an isolate-level bootstrap (5,000 resamples with replacement). This approach rigorously reduces pairwise pseudo replication and serves as an independent validation of the pair-based Hodges-Lehmann (HL) contrasts reported in the main text.

For *L. interrogans*, the median isolate-level spatial shift was 3 SNPs (bootstrap 95% CI: 1-10; n = 20 isolates with ≥1 within-ZCTA comparator), tightly concordant with the pair-based HL contrast reported in the main text (5 SNPs). For *L. borgpetersenii*, the median isolate-level spatial shift was 6 SNPs (bootstrap 95% CI: 4-10; n = 8 isolates with ≥1 within-ZCTA comparator), perfectly matching the pair-based HL contrast (6 SNPs).

**Sensitivity analysis: host structuring (rodents only)**

A parallel per-isolate sensitivity analysis was conducted to validate host-associated genomic structuring among rodents. For each focal isolate $i$, we defined:

- $D_{same}$: The median SNP distance from isolate $i$ to other isolates from the identical host species.
- $D_{other}$: The median SNP distance from isolate $i$ to isolates from different host species.
- $\Delta_{host}=D_{other}-D_{same}$: The additional SNP distance to other-host isolates relative to same-host isolates.

For rodent-derived *L. interrogans*, isolate-level host summaries indicated greater genomic diversity between host species than within hosts, yielding a median shift of 6 SNPs (bootstrap 95% CI: 3-9; n = 20). This outcome strongly supports the significance of the intra- vs. inter-species pair-based HL contrasts reported in the main manuscript.
